# Nutrition delivery of a model-based ICU glycaemic control system

**DOI:** 10.1186/s13613-017-0351-9

**Published:** 2018-01-10

**Authors:** Kent W. Stewart, J. Geoffrey Chase, Christopher G. Pretty, Geoffrey M. Shaw

**Affiliations:** 1 Department of Mechanical Engineering, Centre for Bio-Engineering, University of Canterbury, Private Bag 4800, Christchurch, 8140 New Zealand; 2 Department of Intensive Care, Christchurch Hospital, Christchurch, New Zealand

**Keywords:** Glycaemic control, Nutrition delivery, Clinical workload, Intensive care unit, Critical care, Hyperglycaemia, Hypoglycaemia, Model-based, Targeted

## Abstract

**Background:**

Hyperglycaemia is commonplace in the adult intensive care unit (ICU), associated with increased morbidity and mortality. Effective glycaemic control (GC) can reduce morbidity and mortality, but has proven difficult. STAR is a proven, effective model-based ICU GC protocol that uniquely maintains normo-glycaemia by changing both insulin and nutrition interventions to maximise nutrition in the context of GC in the 4.4–8.0 mmol/L range. Hence, the level of nutrition it provides is a time-varying estimate of the patient-specific ability to take up glucose.

**Methods:**

First, the clinical provision of nutrition by STAR in Christchurch Hospital, New Zealand (*N* = 221 Patients) is evaluated versus other ICUs, based on the Cahill et al. survey of 158 ICUs. Second, the inter- and intra- patient variation of nutrition delivery with STAR is analysed. Nutrition rates are in terms of percentage of caloric goal achieved.

**Results:**

Mean nutrition rates clinically achieved by STAR were significantly higher than the mean and best ICU surveyed, for the first 3 days of ICU stay. There was large inter-patient variation in nutrition rates achieved per day, which reduced overtime as patient-specific metabolic state stabilised. Median intra-patient variation was 12.9%; however, the interquartile range of the mean per-patient nutrition rates achieved was 74.3–98.2%, suggesting patients do not deviate much from their mean patient-specific nutrition rate. Thus, the ability to tolerate glucose intake varies significantly between, rather than within, patients.

**Conclusions:**

Overall, STAR’s protocol-driven changes in nutrition rate provide higher nutrition rates to hyperglycaemic patients than those of 158 ICUs from 20 countries. There is significant inter-patient variability between patients to tolerate and uptake glucose, where intra-patient variability over stay is much lower. Thus, a best nutrition rate is likely patient specific for patients requiring GC. More importantly, these overall outcomes show high nutrition delivery and safe, effective GC are not exclusive and that restricting nutrition for GC does not limit overall nutritional intake compared to other ICUs.

## Background

The ICU patient is under considerable physiological stress, resulting in 20–40% of patients experiencing dysregulation of blood glucose (BG) levels [1] and hyperglycaemia [2, 3], which is associated with increased morbidity and mortality [4–6]. Glycaemic variability due to poor control [7] has also been independently associated with mortality [7–10]. Effective glycaemic control (GC) can reduce mortality and morbidity [11–14], organ failure [15] and cost of care [16, 17]. However, due to inter- and intra- patient variability [18–21], GC has proven difficult, and many protocols have increased hypoglycaemia, also associated with increased mortality [22–25], due to the inability to provide consistent, safe and effective GC [25–31].

The model-based STAR (Stochastic TARgeted) protocol has proven to be safe, consistent and effective [32, 33]. The tablet-based STAR protocol uses a clinically evaluated [34, 35] physiological insulin–glucose model [36, 37] in conjunction with a stochastic model of metabolic variability [38, 39], to estimate a patient-specific current metabolic state and its potential future variability [40, 41]. Thus, treatments are selected by forward simulation with a clinically specified desired risk of light hypoglycaemia (5% BG < 4.4 mmol/L) due to these possible future variations. STAR has proven to be safe, effective and replicable across ICUs [32].

Uniquely, STAR maintains normal BG levels by changing both insulin and nutrition interventions [33]. Changing nutrition interventions differentiates STAR from other ICU GC protocols [42], as most only change insulin interventions (e.g. [43–47]). STAR maximises nutrition in the context of GC in the 4.4–8.0 mmol/L range [33, 40]. Hence, the level of nutrition it provides is a patient-specific, time-varying estimate of the ability to take up glucose and is reduced in the face of significant insulin resistance.

Currently, there is also significant debate over the appropriate amount to feed an ICU patient. Many studies have shown mixed results in reviewing caloric intake, route, and timing and their relation to outcome [48–58]. Cahill et al. [59] surveyed the overall nutrition performance of 158 ICUs, from 20 countries, finding significant variation in nutrition delivery. This study also found an ideal relation to mortality at 85% of the caloric goal nutrition rate set by the respective ICU [51], based on a model fit to the large collection of retrospective survey data obtained from 158 ICUs in 20 countries. This ‘Heyland ideal’ value and the best performing unit surveyed are used in this study as a guideline for assessing the clinical performance of STAR nutrition delivery.

This paper first evaluates the clinical provision of nutrition by STAR, to a cohort of hyperglycaemic ICU patients, versus all ICU patients in other ICUs based on the survey results of Cahill et al. [59] to assess if safe, effective GC precludes or limits high nutrition delivery, as well as determining if nutrition restriction to obtain GC limits total nutritional intake. Second, the inter- and intra- patient variation of nutritional delivery, while maintaining normo-glycaemia, is assessed to evaluate a range of glucose/nutrition tolerance in ICU patients on GC. The main outcomes assess clinically provided nutrition using STAR at the cohort level in an international context and then show a best nutrition rate is likely patient specific, particularly for patients requiring GC.

## Methods

### STAR GC protocol

#### GC protocol overview

Starting criteria for STAR is two successive BG measurements over 8.0 mmol/L within a 4-h period. After two measurements are taken, integral-based parameter fitting [60] is used to identify a clinically evaluated model-based insulin sensitivity [34–36]. This value is used with a stochastic model, based on historical data, [33, 38, 39, 61] to find the 5th and 95th percentile potential future insulin sensitivity values. These 5th and 95th percentile insulin sensitivity values and a potential insulin and nutrition intervention are then used to forward-simulate the likely resulting 5th and 95th percentile BG values for that intervention to find the intervention with 5% risk of BG < 4.4–4.6 mmol/L [33, 40]. Full details can be found in [33].

STAR modifies nutrition rate depending on the bounds of predicted potential behaviour, with a preference to increase insulin before reducing nutrition, and to raise nutrition whenever possible [33, 40]. STAR modulates this nutrition rate between 30 and 100% of the caloric goal, with a maximum step change of ± 30% caloric goal per hour [33]. ACCP guidelines are used to determine patient-specific daily caloric goal intake of 25 kcal/kg/day [62].

Overall, STAR attempts to provide the maximum nutrition rate a patient can tolerate while safely keeping BG in the 4.4–8.0 mmol/L range. However, insulin saturation limits the impact of insulin to lower BG levels on its own [63–65], requiring nutrition restriction in some patients or time periods. Hence, based on STAR’s control predictions, providing excess carbohydrates to a patient above this limit would result in excess BG. Therefore, the nutrition rate achieved by STAR represents a ‘STAR ideal’ patient-specific nutrition rate that maximises their likelihood of falling within the targeted 4.4–8.0 mmol/L BG band, based on their current ability to tolerate glucose.

#### Christchurch clinical implementation

Clinical data from 221 hyperglycaemic ICU patients treated with STAR (2011–2015) [32] in the Christchurch Hospital ICU (mixed medical surgical) were used to assess the performance of its variable nutrition delivery. BG, insulin and nutrition data were collected from STAR tablets and thus only exists when patients are on GC. STAR has proven to provide excellent GC in this cohort spending over 88% time, per patient, in the targeted 4.4–8.0 mmol/L range, as shown in Table 1. STAR patients in Christchurch are typically fed enterally with the low carbohydrate Glucerna™ Select (74.6 g/L Carbohydrate, 50 g/L Protein, 21.1 g/L Fibre, Abbott Labs, Illinois, USA), where carbohydrate concentrations exclude indigestible fibre. Parenteral nutrition (PN) is used occasionally, at clinician discretion, to supplement enteral nutrition. While STAR knows of the PN value, it does not regulate it and will still try to provide 100% of the caloric goal through enteral nutrition (EN). Thus, enabling the possibility of nutrition delivery over 100% of goal. Cohort demographics are given in Table 1.

**Table 1:** STAR cohort patient demographics and GC performance statistics

*Patient demographics*	
Number of patients	221
Number hours of GC	21,769
Age	64.0 [54.0–72.0]
Sex (% Male)	66.1
ICU length of stay	8.4 [3.1–15.3]
Days on GC	2.2 [1.2–3.9]
Admission to GC start (h)	17.5 [7.3–53.8]
Operative (%)	29.0
APACHE II score	21.0 [16.0–27.0]
ICU mortality (%)	28.0
*GC performance statistics*	
BG mean per patient	6.66 [6.36–7.21]
BG SD per patient	1.17 [0.85–1.65]
% Time in targeted band (4.4–8.0 mmol/L) per patient	88.42 [77.42–94.44]
% Time in targeted band (4.4–8.0 mmol/L) cohort	83.2
% Time < 4.4 mmol/L cohort	1.35
# Patients < 2.2 mmol/L	4
Patients fed PN (%)	46.8
Mean days on PN	2.0 [1.0–5.8]
Mean PN per day (% caloric goal)	6.4 [1.5–14.5]

Data presented as median [IQR] where appropriate

Patients are not weighed in the Christchurch ICU, so ACCP caloric goal feed is approximated by estimating the patient weight. This estimation first assumes an 80 kg individual and then modifies this value based on frame size (subjective assessment; small, medium, large), age and sex, using Table 2 and Eq. 1 [66].

1 $$A*F*G*80*25 = {\text{kcal Goa}}l/{\text{Day}}$$

Equation 1 modifies the goal feed rate of 25 kcal/kg/day into a maximum range of 1152–2420 kcal/day. In this cohort, the median interquartile range (IQR) goal feed rate was 1800 [1608–1992] kcal/day. Due to clinical circumstances, such as planned surgery requiring a fasted state, medical imaging, and/or gastric tolerances, a patient’s nutrition may be stopped or reduced significantly, for short periods, not reflective of the STAR feeding algorithm. In this analysis, all occurrences of feeding less than 30% caloric goal are ignored (3,135 h, 14.4% of the time).

**Table 2:** Coefficients used to determine an ICU patients daily caloric goal in Christchurch ICU Hospital

Frame size (F)	Small	Medium		Large
	0.9	1.0		1.1
Age (A)	≤39	40–59	60–79	≥80
	1.1	1.0	0.9	0.8
Gender (G)	Male		Female	
	1.0		0.8	

#### Ethics, consent and permissions

STAR is the standard of care in Christchurch Hospital, New Zealand; therefore, no consent was required from patients to be placed on the STAR GC protocol. The Upper South Regional Ethics Committee, New Zealand, granted approval for the audit, analysis and publication of the retrospective data.

### Analysis

#### Overall clinical performance of current STAR variable nutrition protocol

The mean cohort caloric goal achieved per day in the ICU by STAR, with hyperglycaemic ICU patients, is calculated and compared to the entire ICU patient cohorts reviewed by Cahill et al. [59]. For STAR, information only exists for periods of GC, which are aligned to the appropriate day of ICU stay so comparisons to Cahill et al. [59] are valid. The percentage of caloric goal achieved represents the total caloric intake (including protein calories) from both EN and PN, in regard to the ACCP caloric goal. This analysis helps answer whether caloric restriction for GC, or safe, effective GC in general, preclude or limit nutrition delivery when compared to that achieved by the entire ICU patient cohort.

#### Per-patient nutrition delivery

The distribution per patient (median, IQR, 5th–95th range) of caloric goal achieved per day on STAR is calculated. The per-day distribution is compared to the best performing ICU surveyed in [59] and the 85% ‘Heyland ideal’ caloric goal presented in [51] to evaluate the percentage of patients who can tolerate more, or less, nutrition than these results. This comparison delineates the range and distribution of glucose and nutrition tolerance for these medical ICU patients.

The mean and variation of caloric goal achieved over a patient’s entire stay is assessed in terms of median IQR between patients and to the overall variation seen per day across the entire cohort. This assesses if the overall variability seen per day is due to variable patients or different patient-specific tolerances of nutritional uptake.

## Results

### Overall clinical performance of current STAR variable nutrition protocol

**Fig. 1:**
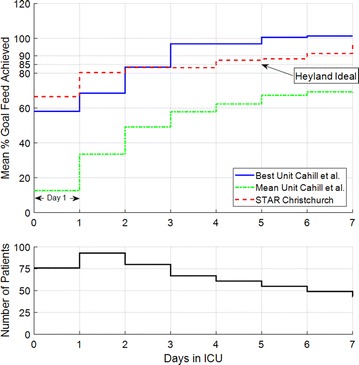
(Top) Comparison of mean percentage goal feed achieved for each day in the ICU between STAR Christchurch clinical results and the results published in Cahill et al. The ‘Heyland ideal’ 85% caloric goal, to minimise mortality, presented in Heyland et al. [51] is also provided for comparison. (Bottom) The number of patients per day, where it is important to note that not all of the 221 patients start on day 1

The percentage caloric goal clinically achieved by STAR, each day in ICU, was compared to the survey results in Cahill et al. [59]. Figure 1 shows mean nutrition delivered to hyperglycaemic ICU patients by the variable nutrition protocol in STAR performs very well compared to all ICU patients in the best ICU reviewed in Cahill et al., only slightly underperforming after day 3. It is well above the mean ICU surveyed on all days, as shown in Table 3. In addition, the mean percentage caloric goal nutrition, per day in ICU, exceeds the ‘Heyland ideal’ 85% caloric goal [51] from day 4 onwards.

**Table 3:** Percentage of patients above the Mean ICU reviewed by Cahill et al. [59] and ‘Heyland ideal’ rate of 85% [51]

Day in ICU	Day 1	Day 2	Day 3	Day 4	Day 5	Day 6	Day 7
% Patients > Mean Unit Cahill et al.	100.0	96.8	92.5	86.6	85.3	90.9	85.7
% Patients > ‘Heyland ideal’ (85%).	25.0	41.9	56.2	58.2	63.9	60.0	73.5

### Per-patient nutrition delivery

**Fig. 2:**
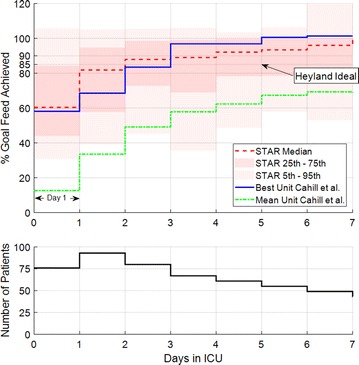
(Top) Comparison of the median interquartile range of STAR Christchurch’s percentage goal feed achieved clinically (*N* = 221 Patients) and the best performing unit reviewed in Cahill et al. The ‘Heyland ideal’ 85% caloric goal, to minimise mortality, presented in Heyland et al. [51] is also provided for comparison. (Bottom) The number of patients per day, where it is important to note that not all of the 221 patients start on day 1

Figure 2 shows the distribution of per-patient mean nutrition rates delivered per day by STAR, including IQR and 5th–95th percentile values. It clearly shows large variation in patient-specific nutrition rates on the first day of ICU stay, which narrows as patient-specific metabolic state stabilises [21]. Table 3 shows over 56.2% of patients reach or exceed the ‘Heyland ideal’ 85% caloric goal in [51] after day 2, reaching 73.5% on day 7. The percentage of patients over the mean ICU result in [59] are also shown in Table 3 to be ranging from 100% on day 1 to 85.7% on day 7. Overall, in comparison with Fig. 1, the per-patient results clearly show some patients cannot achieve this cohort mean rate or the ideal 100% caloric goal. As noted, the rates in Fig. 2 are an estimate of the ‘STAR ideal’ time-varying patient-specific nutrition uptake in the context of GC to the 4.4-8.0 mmol/L BG range.

Table 4 shows the median of the mean feed rate achieved over a patient’s stay, per patient, is relatively high at 89.8% caloric goal, but has a large IQR of 23.9%. However, the relatively small median standard deviation of feed rate achieved over a patient’s stay, per patient, of 12.9% shows that individual patients are less variable than the cohort and thus that the overall ability to tolerate glucose is patient specific. Thus, it is clear the ability to take up, and thus to deliver, nutrition varies significantly between GC patients.

**Table 4:** Per-patient feed rate characteristics

Number of patients	221
Mean of a patient’s feed rate over entire stay, per patient (%)	89.8 [74.3–98.2]
SD of a patient’s feed rate over entire stay, per patient (%)	12.9 [4.6–20.4]

Data presented as median [IQR] where appropriate

## Discussion

### Overall clinical performance of current STAR variable nutrition protocol

Figure 1 shows STAR’s nutrition protocol, on hyperglycaemic ICU patients, performs equal to or better than the average of all the ICU patients in the best ICU surveyed by Cahill et al. [59] over the first 3 days of ICU stay. After day 3, the best ICU performs slightly better. However, the number of patients on GC is shown to diminish after day 3. This outcome makes the relevance of nutrition performance less significant after this time. Overall, these outcomes show the current STAR nutrition protocol delivers clinical nutrition results for hyperglycaemic patients, which are equal to, or better than, those reported in the Cahill et al. survey for all ICU patients in 158 ICUs in 20 countries. It is clear that high nutritional delivery and safe, effective GC are not mutually exclusive and that variable nutrition to achieve GC does not reduce total nutritional intake when compared to an entire ICU cohort.

### Per-patient nutrition delivery

Figure 2 shows a large variation in nutrition rates achieved per day, per patient, narrowing and rising as the patient-specific metabolic state stabilises [21], similar to that seen in Heyland et al. [67]. However, the median variation per patient was only 12.9 [4.6–20.4] % (Table 4), suggesting patients do not deviate significantly from their mean nutrition rate. This result and the large IQR of the mean feed rates achieved (74.3–98.2%, Table 4) suggest the lower nutritional delivery to the 5th and 25th percentile are a result of a few patients who had a lower ability to tolerate glucose intake.

It is very uncommon for patients not on GC in Christchurch ICU to have their feed rates changed due to the strong clinical culture of patients achieving their caloric goal. In addition, prior to STAR’s predecessor, SPRINT, being implemented (2005) [14], feed rates were fixed at 100% caloric goal during GC for all patients. Hence, if they are not on GC, they are likely to have a fixed 100% caloric goal nutrition rate and have a BG within 4.4–8.0 mmol/L, having a relatively constant glucose tolerance and reduced insulin sensitivity variability [21, 68]. Therefore, if all patient data were considered, the intra-patient variability seen in Table 4 would likely go down.

Considering STAR feeds the maximum possible nutrition, while safely maintaining normo-glycaemia, the nutrition rates achieved give a good indication of the patient-specific ability to tolerate glucose and thus of their ‘STAR ideal’ nutrition rate. In essence, every patient is fed the maximum they can achieve with added insulin, within the bounds of the future predicted variability. Therefore, the spread of nutrition rates per patient in the results infer this ‘STAR ideal’ nutrition rate is very patient specific and evolves with time.

The ‘STAR ideal’ nutrition rate achieved by STAR was less than the 100% caloric goal for more than 50% of patients, over all days. However, the best unit surveyed in Cahill et al. [59] was still considerably lower than this predetermined caloric goal suggesting these generalised approximations do not represent all ICU patients well, as seen in the results for STAR in Christchurch. In addition, over 56% of patients exceeded the lower 85% ‘Heyland ideal’ of [51] by day 3, as shown in Table 3.

### Limitations

Cahill et al. [59] provides the percentage caloric goal nutrition achieved by each ICU. However, caloric goals may vary across ICUs. Additionally, the estimation of patient body weight in Christchurch ICU [69], as shown in Table 2, may also bias the caloric goal feed estimate which outlines the need for patients to be weighed on the day of ICU admission in Christchurch Hospital. As a result, some ICUs may thus achieve caloric goal nutrition targets ‘more easily’ than others, making comparison difficult. However, the 25 kcal/kg/day ACCP guideline [62] used in the Christchurch ICU, or a similar value guideline (25–30 kcal/kg/day SCCM/ASPEN [70], and 20–25 kcal/kgBW/day initial phase and recovery phase 25–30 kcal/kgBW/day ESPEN [71]), is commonly used and these cover the range used with STAR patients.

In addition, Cahill et al. review nutrition achieved during the first day of ICU stay, which is not necessarily when GC starts for all patients. Although GC commonly starts at the beginning of ICU stay, it may not always be the case. However, as an ICU patient is under the most amount of stress immediately post-surgery or insult [72], they are most likely to require GC at or near the beginning of their ICU stay [1–3]. In this study, 59.3% of patients started GC within 24 h of being admitted to the ICU (Median 15.5 h, Table 1).

Moreover, Cahill et al. survey the nutrition given to all ICU patients. However, this study only considered patients who required GC. The 25–35% of patients who require GC in the ICU [28] are the most metabolically stressed and, as a result, have a reduced glucose uptake capacity. They are thus often harder to deliver the target nutrition rates [20, 52, 57, 58]. In addition, given that 158 ICUs over 20 countries were surveyed by Cahill et al. [59], and this ICU was 1 of the 22 surveyed in Australia, and New Zealand the mixed medical surgical ICU in Christchurch Hospital would likely have patients with similar parameters. Therefore, achieving nutrition rates with high performance GC similar to that achieved for all ICU patients, normo-glycaemic and hyperglycaemic, in the best ICU reviewed by Cahill et al. [59] is a significant outcome. More importantly, this outcome and the inter-patient variability in the results indicate high nutrition delivery and safe, effective GC are not exclusive, an equally, that nutrition restriction to obtain GC does not necessarily reduce total nutrition in an international context.

The insulin–glucose model used by STAR has been shown to be effective in predicting a patient’s response [35, 36, 73]. However, as STAR doses based on the 5th and 95th percentile future metabolic variability [38], ensuring only a 5% risk of hypoglycaemia [74], the majority of patient’s future BG will fall within the targeted band. Hence, many patients could possibly remain within the targeted BG range (4.4–8.0 mmol/L) if given a higher than recommended nutrition rate. As a result, some patients may be able to receive higher nutrition rates than reported here and still be able to be provided effective GC. However, this choice would also increase the likelihood of hyperglycaemia, reducing the safety of GC provided by STAR.

The 85% caloric goal presented in Heyland et al. is calculated by a model fit to retrospective data from 158 ICUs and clinical practices, and while it represents a significant body of multi-centre data, it may not be causative. Many prospective trials have found improved outcomes for even lower hypo-caloric feeding [50, 75–77]. Therefore, this ‘Heyland ideal’ value may overestimate the caloric goal required for improved outcomes and may be reflective of ‘less sick’ patients tolerating higher nutrition. This study is designed to show that STAR can provide high nutrition rates while still providing safe and effective GC. In addition, STAR is designed to be flexible to different nutrition goals while still providing effective GC.

Other factors, such as mechanical ventilation, neurologic injury, gastric emptying and paresis patients, are well known to influence the nutritional requirements of ICU patients. This is another strong limitation of this retrospective analysis, as this detailed information was not available. However, the cohort was typical of medical ICU in Christchurch.

The STAR GC protocol uses model-based patient-specific control in conjunction with a stochastic model to predict the best treatment for a patient. As shown in Table 1 and [32], STAR is able to achieve very good GC with a compliance of over 96.8% in all interventions and near identical results across multiple ICUs [32]. However, in many clinical practices, the idea of protocol-driven changes in the nutrition given to a patient for GC is foreign and thus clinically unacceptable. Thus, the main focus of this study is to show that protocol-driven changes in nutrition rate do not preclude in achieving better nutrition delivery rates than those of 158 ICUs from 20 countries. In addition, the concept of nutritional tolerances in relation to glucose tolerances provides a potentially new method of calculating patient-specific feed rates and should be investigated further in future studies.

## Conclusions

The STAR GC protocol clinical provision of nutrition to hyperglycaemic patients was compared to nutrition rates of entire ICU cohorts surveyed in 158 ICUs in Cahill et al. [59]. Mean nutrition rates clinically achieved by the STAR variable nutrition protocol were significantly higher than the mean and best ICU surveyed, for the first 3 days of ICU stay. Overall, STAR’s protocol-driven changes in nutrition rate provide on average nutrition rates for hyperglycaemic patients which are equal to, or better than the mean of all ICU patients in 158 ICUs from 20 countries. More importantly, these outcomes show high nutrition delivery and safe, effective GC are not exclusive and that restricting nutrition for GC does not limit overall nutritional intake compared to other ICUs.

The inter- and intra- patient variation of nutritional delivery was assessed in the STAR cohort There was large inter-patient variation in nutrition rates achieved per day, which reduced overtime as patient-specific metabolic state stabilised. Median intra-patient variation was 12.9%; however, the IQR of the mean per-patient nutrition rates achieved was 74.3–98.2%, suggesting patients do not deviate much from their mean patient-specific nutrition rate and thus that the ability to tolerate glucose intake varies significantly between, rather than within, patients. There is significant inter-patient variability between patients to tolerate and uptake glucose, where intra-patient variability over stay is much lower. Thus, a best nutrition rate is likely patient specific for patients requiring GC.

## Abbreviations

STAR: stochastic TARgeted
GC: glycaemic control
BG: blood glucose
IQR: interquartile range
ICU: intensive care unit
ACCP: American College of Chest Physicians
PN: parenteral nutrition
